# Application of verbal autopsy in routine civil registration in Lusaka District of Zambia

**DOI:** 10.1186/s12913-021-06427-y

**Published:** 2021-05-01

**Authors:** C. Chabila Mapoma, Brian Munkombwe, Chomba Mwango, Bupe Bwalya Bwalya, Audrey Kalindi, N. Philimon Gona

**Affiliations:** 1 Department of Population Studies, School of Humanities and Social Sciences, University of Zambia, Lusaka, Zambia; 2 International Statistics Program, Centre for Disease Control and Prevention, Lusaka, Zambia; 3 Bloomberg Data for Health Initiative, Lusaka, Zambia; 4 Department of Economics, Directorate of Research and Postgraduate Studies and School of Social Sciences, Mulungushi University, Main Campus, P.O. Box 80415, Kabwe, Zambia; 5 College of Nursing and Health Sciences, University of Massachusetts Boston, 100 Morrissey Boulevard, Boston, MA 02125 USA

**Keywords:** Cause of death, Verbal autopsy, SmartVA

## Abstract

**Background:**

Ascertaining the causes for deaths occurring outside health facilities is a significant problem in many developing countries where civil registration systems are not well developed or non-functional. Standardized and rigorous verbal autopsy methods is a potential solution to determine the cause of death. We conducted a demonstration project in Lusaka District of Zambia where verbal autopsy (VA) method was implemented in routine civil registration system.

**Methods:**

About 3400 VA interviews were conducted for bodies “brought-in-dead” at Lusaka’s two major teaching hospital mortuaries using a SmartVA questionnaire between October 2017 and September 2018. Probable underlying causes of deaths using VA and cause-specific mortality fractions were determined.. Demographic characteristics were analyzed for each VA-ascertained cause of death.

**Results:**

Opportunistic infections (OIs) associated with HIV/AIDS such as pneumonia and tuberculosis, and malaria were among leading causes of deaths among bodies “brought-in-dead”. Over 21.6 and 26.9% of deaths were attributable to external causes and non-communicable diseases (NCDs), respectively. The VA-ascertained causes of death varied by age-group and sex. External causes were more prevalent among males in middle ages (put an age range like 30–54 years old) and NCDs highly prevalent among those aged 55 years and older.

**Conclusions:**

VA application in civil registration system can provide the much-needed cause of death information for non-facility deaths in countries with under-developed or non-functional civil registration systems.

## Introduction

Accurate causes of death are necessary to help better understand the patterns and major causes of mortality of a country [1]. When monitored over time, cause of death (COD) information can be useful in tracking population-level trends and patterns in mortality, which are crucial for health and development policies, health programs; program monitoring and evaluation [2]. However, many developing countries such as Zambia, with among the highest burden of disease, do not have reliable cause of death information. There is lack of robust and reliable civil registration and vital statistics (CRVS) systems to generate reliable, representative and high quality national or sub-national causes of death [3, 4]. In addition, a large proportion of the deaths in these countries occur outside health facilities where there is no doctor present to certify such deaths. Ad-hoc studies and surveys are useful alternatives to fill the information gaps, they however are not sustainable since they depend largely on the availability of external technical and resources and funding.

A complete diagnostic medical-pathology autopsy is the most reliable method available for ascertaining cause of death. Such systems, however, are neither practical or attainable in many (developing) countries. Minimally invasive tissue sampling is an alternative approach that provides similar validity for ascertaining causes of deaths but this high-tech approach requires availability of skilled health personnel and well-equipped laboratories [5]. Both pathology diagnostic autopsy and minimally invasive tissue sampling are the most common modalities for ascertaining cause of death in health facilities [6]. However, these techniques require substantial resources, personnel, and appropriate infrastructure, a medical certification of cause of death, through the WHO’s International Classification of Diseases (ICD-10), is the most common routine to ascertain the cause of death in health facilities [6]., A majority of deaths in many developing countries occurr outside health facilities and are not attended to or certified by medical practitioners [7]. Verbal autopsy (VA) is, therefore, a more practical method for ascertaining causes in places where a large proportion of deaths occur outside of the health facilities and where systems for medical certification of causes of death are weak [4, 8].

In Zambia, mortality records are collected from health facilities throughout the country and reported to the Department of National Registration Passports and Citizenship (DNRPC). However, most deaths occurring outside health facilities are not captured in the registry as they not reported. Previous surveys indicate that 48% of deaths in Zambia occur outside health facilities [9, 10]. In the Zambia capital city of Lusaka, as in other urban areas, non-facility deaths are brought to the hospital mortuaries as “brought in dead” (BID). However, most of these deaths are not recorded in the health information systems therefore they are not reported in civil registration systems [11]. Although some BIDs have accompanying police reports on which causes of deaths is usually recorded, most of these police indicated causes of death, especially for natural deaths, are very inaccurate, unreliable and are not validated, rendering them unusable for public health policy and decision-making. Therefore, implementing the VA to ascertain causes of death is a more practical solution [12] with greater utility for countries such as Zambia which still has to develop her CRVS. In addition, given the underreporting of certain causes of death such as HIV-associated deaths or related diseases or certain (presumably) stigmatized deaths such as death by suicide, VA may improve help to the validity of non-facility mortality estimates [13].

Verbal autopsy is a validated method for determining causes of death in populations without a complete vital registration system. Verbal autopsies consist of a trained interviewer using a questionnaire to collect information about the signs, symptoms, and demographic characteristics of a recently deceased person from an individual familiar with the deceased [8, 14, 15]. VA is based on the assumption that most causes of death can be distinguished by their signs and symptoms and that these can be accurately recognized, recalled and reported by lay respondents [16]. At aggregate level, the VA is the best available method for providing cause of death information for non-facility deaths. VA can be applied in routine settings, such as the civil registration and vital statistics, to generate robust national and subnational cause of death data for deaths that occur outside the health facility and without the benefit of medical certification of cause of death [17, 18]. In addition, the VA information can complement information on causes of death generated from health facilities, thereby presenting a more complete picture of the pattern of cause of deaths in a particular area or domain of analysis. The VA approach is used by large networks such as the Global Burden of Disease (GBD) [15, 19] and Demographic Health Surveys (DHS) [20, 21],

Understanding causes and characteristics of death occurring outside health facilities is important as they serve as an important source of information for population health monitoring, priority setting and planning [22]. Thus helping experts in the health sector coming up with public health policies and making decisions that responds to such deaths, since these deaths might be different (in characteristics) from those that occur in health facilities. This paper reports findings from the current Zambia VA application. Details of reported information includes leading causes of non-facility deaths in Lusaka (in the data collection period) further analyzed by demographic characteristics of the deceased with broad categories of cause of death This paper however present just a subset of the mortality profile of Lusaka because health facility deaths were not included..

## Methods

Data was obtained from VA application in routine civil registration of BID cases conducted by the Ministry of Home Affairs’ Department of National Registration Passports and Citizenship (DNRPC). The DNRPC is responsible for birth and death registration in Zambia. The DNRPC, with technical support from the Bloomberg’s Data for Health (D4H) initiative, has implemented CRVS improvement activities in Zambia to improve its death registration completeness. The target population was all BIDs brought through to mortuaries of the two major hospitals in Lusaka, namely the University Teaching Hospital (UTH) and Levy Mwanawasa Hospital. The period covered was from October 1st, 2017 through September 30st, 2018.

Deaths that occur outside health facilities in Lusaka are usually reported first to the police, who certify the death as either naturally, or unnaturally occurring and thereby providing the family with a police report required for registering the deaths and obtaining a death certificate. The family then uses this police report to obtain a burial order or cremation permit. Since traditional funeral rituals are generally held for 2 or 3 days after death, the deceased bodies are usually brought to the hospital mortuaries or morgues for storage while the funeral ceremonies are ongoing. The DNRPC and D4H Initiative supports registry clerks who register BIDs and administer a VA interview to family members of the deceased.

Although over 90% of deaths occurring in Lusaka district are ably captured by the CRVS VA. There is still a 10% which go uncaptured. This is despite the law prohibiting registration or disposing off a body without cause of deaths information).

A three-module streamlined or Smart VA questionnaire developed and validated by the population health metrics research consortium (PHMRC) was used. The three modules included theneonate questionnaire for infants who were born alive but died within 28 days. The Child questionnaire for children who died between 28 days and 12 years of age, and the Adult questionnaire for all deceased individuals aged 12 years and older. The instrument was implemented electronically on an open data kit (ODK) collection system using android tablets. Using tablets saves time while reducing interviewer data entry errors thereby enhancing the quality of completed questionnaires [23].

After each interview, the interviewer uploaded the data of the completed questionnaire to ODK Aggregate, a ready to deploy server that is available with the ODK program. The data was validated through the application’s in-built edit and logical checks. Records with errors were sent back to interviewers for editing and correction. Completed interviews were run through a SmartVA-Analyze application. The SmartVA-Analyze was developed by the Institute for Health Metrics and Evaluation (IHME) at the University of Washington, Seattle Washington. SmartVA-Analyze allows the application to implement computer certification of verbal autopsies using the Tariff Method [19]. The Tariff Method processes and produces causes of death (COD) estimates at both individual and population/aggregate levels for 34 adult causes, 21 child causes, and six neonatal causes [23]. Algorithms built in SmartVA-Analyze use probabilistic modelling techniques to determine the likely COD(s) for each VA case; the model workings are based on machine-learning trained using a combination of expert medical opinion and relevant available data [20, 24].

For a city such Lusaka with a high prevalence of HIV and a low prevalence of malaria (settings recommended by IHME) the SmartVA-Analyze was set to ‘low’ malaria and ‘high’ HIV/AIDS. Although Zambia is a high malaria endemic as a country, Lusaka has low malaria risks. This assertion is supported by Masaninga et al’s study which reported that Lusaka has very low transmission rates of malaria compared with other parts of the country [25].

The Tarrif algorithm was applied to each case, yielding a probable underlying cause of death for each VA. In the few cases where symptoms were vague, contradictory or mutually inconsistent, Smart VA was unable to determine cause of death and as result, these deaths were deemed indeterminate [19]. Using the generated probable underlying causes of death for each VA, Tarrif calculated Cause Specific Mortality Fractions (CSMFs) for each cause for the three questionnaire modules: Neonate, Child and Adult.

The probable causes of death data derived from the SmartVA-Analyze were exported from ODK Aggregate to Microsoft Excel for the purpose of analyzing causes of death by age and sex patterns. Cause of death data were grouped using SPSS statistical software into three broad Global Burden of Disease (GBD) cause categories namely: communicable, non-communicable, and external (injury and accidental) causes; This three-level COD classification was used as the dependent variable in statistical analyses. Demographic characteristics (age and sex,) were stratified by the COD categories. A Chi-squared test was used to examine the associations between the demographic characteristics (age and sex) and the COD outcomes. Since the dependent variable had three possible discrete outcomes multivariable multinomial logistic regression [26, 27], with the communicable disease as the referent category, was used to model the log-odds associated with the demographic variables.

The Zambian Government’s civil registration authority and the Bloomberg D4H sanctioned the implementation of the VA in 2015. Since verbal autopsy required interviewing the next of kin of the deceased, an ethical clearance was obtained through the University of Zambia Biomedical Research Ethics Committee. Written informed consent was also obtained from each interviewee before proceeding with the interview.

## Results

### General death distribution

From the two major hospitals in Lusaka: UTH and Levy Mwanawasa hospital mortuaries, 3411 VA interviews were conducted (see Table 1) of which, 154 (4.5%) were from the neonate questionnaire (less than 28 days), 436 (12.8%) were from the child questionnaire (28 days to 11 years) and 2821 (82.7%) were from the adult questionnaire (12 years and older). At least 64% (2179) of the males participated in the VA interviewed compared to 36% (1232) of the females. Infant deaths (< 1 year) were 351 (10.3%), while those aged 1–4 and 5–19,190 (5.6%) and 206 (6.0%), respectively. Majority of deaths occurred in the age group 35–54 years that accounted for 1040 (30.5%) of all the VAs. External (injury and accidents) accounted for 27.5% of all male deaths compared to only 11% for female deaths. The largest proportion of the deaths due to the communicable diseases were among the infants and 1 to 4 years while the deaths due to non-communicable diseases were highest among those 55 years or older). External causes (Injury and Accidents) were highest among 5–19 and 20–34 years Age groups.

**Table 1:** Proportion of the Demographic Characteristics of Verbal Autopsy Interviews Stratified by Broad Cause of Death Categories (*n* = 3411)

			Broad Causes of Death Categories		
Characteristics	Number	Communicable	Non-Communicable	External (Injury and Accidents)	Undetermined
	n	%	%	%	%
**Sex**					
Male	2179	34.5	22.7	27.5	15.3
Female	1232	39.0	34.3	11.0	15.7
Total	3411	36.1	26.9	21.6	15.5
**Age Groups**					
0	351	61.0	17.1	0.3	21.7
1–4	190	52.6	4.2	20.0	23.2
5–19	206	27.7	11.7	46.6	14.1
20–34	761	32.1	15.0	38.2	14.7
35–54	1040	44.5	18.9	22.9	13.7
55+	830	18.3	61.6	5.7	14.5
Missing Age	33	6.1	6.1	75.8	12.1
Total	3411	36.1	26.9	21.6	15.5

### Distribution of deaths by age and sex

**Fig. 1:**
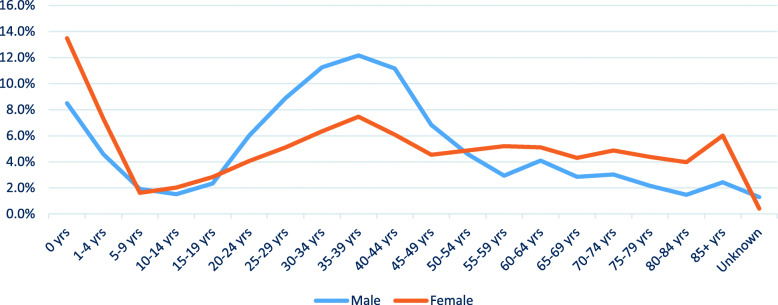
Percent Distribution of the Non- Facility Deaths by Age and Sex

Age-sex distribution (shown in Fig. 1) of deaths is expected for a developing country such as Zambia where mortality in the early years after birth is high; declining in childhood and teenage years and thereafter increases between ages 20–49 years (with higher levels of mortality for males than females) and later declining to stable levels in older ages (with higher levels for females than males).

### Distribution of probable causes of death

**Fig. 2:**
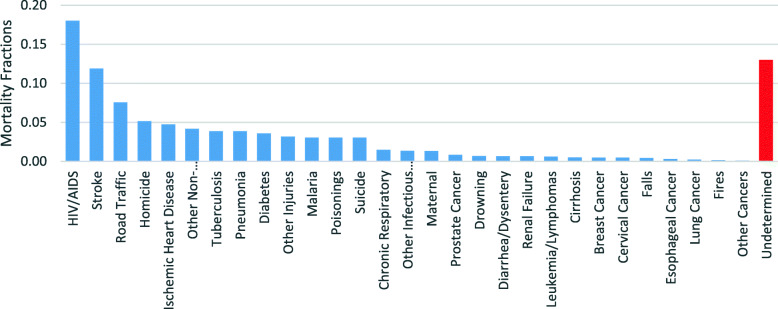
Adult Cause Specific Mortality Fractions

Cause specific mortality fractions for adult deaths (in Fig. 2) show HIV/AIDS related deaths, stroke, road traffic accidents, homicide, ischemic heart diseases, pneumonia, tuberculosis, diabetes and malaria as leading causes of non-facility deaths in Lusaka, with causes such as prostate cancer, breast cancer, lung cancer, cirrhosis and others also contributing to the burden of adult deaths. HIV/AIDS related deaths contributed 18% of all non-facility adult deaths, with stroke (at 12%) and road traffic accidents (at 8%) as second and third most contributors respectively.

**Fig. 3:**
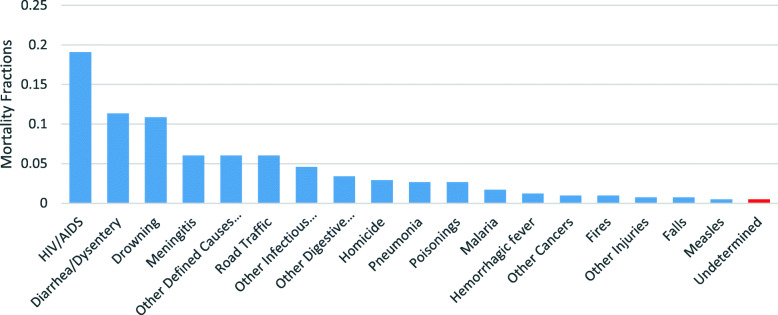
Child Cause Specific Mortality Fractions

For deaths occurring in children (aged 28 days to 11 years), HIV/AIDS related death was the leading cause accounting for 19% of all non-facility deaths in children. Diarrhea/Dysentery (at 13% and drowning at 10% (Fig. 3). Other causes such as road traffic accidents, malaria, measles and accidental falls also contributed to the burden of child deaths dying outside the health facilities.

**Fig. 4:**
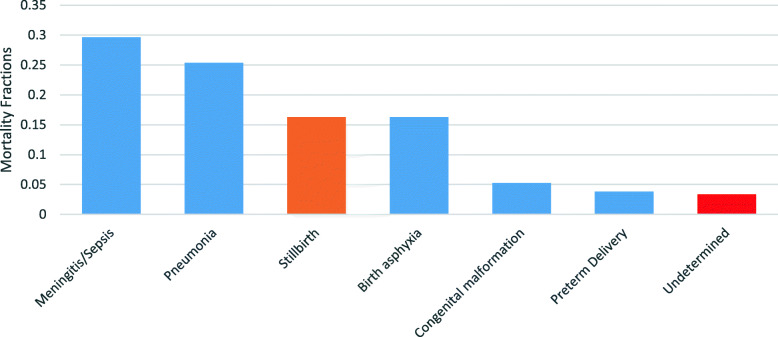
Neonate Cause Specific Mortality Fractions

Among neonatal deaths, meningitis/sepsis was the leading cause accounting for 30% of all non-facility neonatal deaths (Fig. 4); Pneumonia (at 25%) and birth asphyxia (at 16%) were the second and third leading causes of neonatal deaths for the period under review. Other causes such as preterm delivery and congenital malformation were also substantial contributors to the burden of neonatal deaths.

### Classification of causes of death into three broad global burden of disease groups

Causes of death were categorized into three broad cause groups following the pattern of the GBD: these three broad categories include communicable diseases (CDs), non-communicable diseases (NCD) and external causes (injuries and accidents). Results in Table 2 show that for the period under review, communicable diseases were the leading causes of deaths in Lusaka (36%) followed by non-communicable (27%) and external causes (22%) respectively.

**Table 2:** Proportion of the Main Leading Causes of Death grouped in the Three Broad Cause Groups: Communicable, Non-Communicable and External Causes and Age Group

Type of Cause of Death	Number	< 1 Yr	1–4 Yrs	5–19 Yrs	20–34 Yrs	35–54 Yrs	55+ Yrs
Communicable, Maternal	1230		%	%	%	%	%
HIV/AIDS related deaths	568	3.2	8.3	3	21.8	54.9	8.8
Pneumonia	152	19.1	9.9	3.9	16.4	28.3	22.4
Tuberculosis	115	0	0	2.6	28.7	34.8	33.9
Malaria	89	1.1	4.5	6.7	24.7	40.4	22.5
Diarrhea/Dysentery	63	9.5	58.7	9.5	1.6	12.7	7.9
Meningitis/ Sepsis	53	100	0	0	0	0	0
Other Infectious Diseases	52	0	25	7.7	34.6	25	7.7
Maternal	37	0	0	13.5	56.8	29.7	0
Stillbirth	34	100	0	0	0	0	0
Meningitis	25	24	72	4	0	0	0
Other Defined Causes of Death	42	38	40	22	0	0	0
**Non-communicable Diseases**	**914**						
Stroke	328	0	0	2.4	9.8	18.6	69.2
Ischemic Heart Disease	131	0	0	0	14.5	21.4	64.1
Other Non-communicable Diseases	115	0	0	4.3	30.4	34.8	30.4
Diabetes	99	0	0	0	11.1	24.2	64.7
Other Cardiovascular Diseases	50	72	12	10	2	0	4
Chronic Respiratory	41	0	0	0	7.3	26.8	65.9
Prostate Cancer	23	0	0	0	4.3	0	95.7
Renal Failure	18	0	0	0	22.2	33.3	44.4
Leukemia/Lymphomas	17	0	0	5.9	23.5	41.2	29.4
Cirrhosis	14	0	0	0	7.1	50	42.9
Breast Cancer	13	0	0	0	7.7	46.2	46.2
Cervical Cancer	13	0	0	0	0	23.1	76.9
Other Digestive Diseases	12	41.7	33.3	25	0	0	0
Congenital malformation	8	100	0	0	0	0	0
Other Cancers	25	0	8	8	8	16	60
Preterm Delivery	7	100	0	0	0	0	0
**External Causes**	**711**						
Road Traffic	227	0	2.6	11.9	37.9	36.1	11.5
Homicide	153	0	5.9	8.5	47.1	32	6.5
Poisonings	91	0	4.4	9.9	51.6	31.9	2.2
Other Injuries	91	0	2.2	13.3	35.6	44.4	4.4
Suicide	84	0	0	14.3	48.8	32.1	4.8
Drowning	44	0	34.1	38.6	15.9	9.1	2.3
Falls	14	0	0	28.6	21.4	50	0
Fires	7	0	42.9	14.3	42.9	0	0
**Undetermined Causes**	523	11.9	11.1	5.5	21.4	27.2	22.9
**Missing Age**	**33**						

Leading causes of death under communicable diseases included; HIV/AIDS related deaths, pneumonia, tuberculosis, malaria and diarrhea/dysentery (see Table 2). Disaggregated by age, HIV/AIDS related deaths were highest between 20 and 54 years of age (over 76%). Pneumonia related deaths were higher among infants (19%) and those aged 35 years and older (51%). In the same way, tuberculosis affected mainly those in the age groups 20 and 54 years as well those in older age groups (55+ years). The burden of Malaria was highest among the same age groups (20–54), while Meningitis/sepsis affected infant more; diarrhea/dysentery on the other hand was more prominent in children under 5. Maternal deaths were more prominent among women aged 20–34 years.

As for NCDs, stroke, ischemic heart disease, diabetes and other cardiovascular diseases were leading causes for death in Lusaka for the period under review. Most NCD deaths occurred in older age groups. For instance, 70% of those who died from stroke were aged 55 years or older; over 64% of those who died from ischemic heart disease were aged 55 years or older. This pattern was observed for all other non-communicable diseases – that is, affecting mostly older age groups- except for other cardiovascular diseases, preterm delivery and congenital malformation that (also) affected infants.

Leading causes of deaths classified as externally caused were road traffic accidents, homicides, poisoning and suicides. The most affected age range for externally caused deaths was between 20 and 54 years, however, majority of drownings and fire related deaths occurred in children and young adults of less than 20 years old.

### Assessing causes of death by age and sex

Table 3 shows that. Males had a significantly higher proportion (32%) of deaths due to external causes than females (at 13%), *p* < 0.0001. Females had, on the other hand a significantly higher proportion (41%) of deaths due to NCDs (especially occurring in the older age group of 55+ years) than males (at 27%). The proportion of deaths due to communicable diseases were not materially different between males and females.

**Table 3:** Proportion of the Broad Causes of Death Categories stratified by Age and Sex

		External (Injury and Accidents)	Non-Communicable	Communicable	Number
**Sex**	**Age Groups**				
**Male**	0	0.0	25.7	74.3	113
	1–4	22.9	6.7	70.5	105
	5–19	61.5	9.6	28.8	104
	20–34	52.4	16.9	30.6	490
	35–54	31.3	19.3	49.5	649
	55+	8.3	66.4	25.3	360
	**Total**	**31.7**	**27.1**	**41.2**	**1821**
**Female**	0	0.0	25.5	74.5	106
	1–4	15.5	5.2	79.4	97
	5–19	48.3	19.2	37.0	73
	20–34	21.4	19.5	59.1	159
	35–54	14.1	28.9	57.0	249
	55+	4.9	77.7	17.4	350
	**Total**	**12.9**	**40.7**	**46.4**	**1034**
**Chi-Squared Tests**					
Male *p* < 0.0001	Female *p* < 0.0001				

Results of the multivariable adjusted multinomial regression are shown in Table 4: In Model 2, age-group 55+ years was the referent category; and female was used as reference for the sex variable. Akaike Information Criterion (AIC) was used to assess model fit whereby a model having a lower AIC value represents a better fit.

**Table 4:** Multinomial Regression of the Cause of Deaths Categories by Age Group and Sex

Cause of Death (Ref = Communicable)		Model 1 Odds Ratio	95% CI		Sig. (***p*** = 0.05)	Model 2 OR	95% CI		***P***-value
			Lower Bound	Upper Bound			Lower Bound	Upper Bound	
**Non-Communicable**	**Sex (Ref = Female)**								
	Male	0.79	0.66	0.94	**0.01**	0.86	0.71	1.05	0.14
	**Exact Age**	1.02	1.02	1.02	**0.00**				
	**Age Group (Ref = 55+ Years)**								
	0					0.10	0.07	0.15	**0.00**
	1–4					0.02	0.01	0.04	**0.00**
	5–14					0.12	0.08	0.21	**0.00**
	15–34					0.14	0.11	0.19	**0.00**
	35–54					0.13	0.10	0.17	**0.00**
**External Causes**									
	**Sex (Ref = Female)**								
	Male	2.96	2.37	3.70	**0.00**	2.56	2.02	3.24	**0.00**
	**Exact Age**	1.02	1.02	1.02	**0.00**				
	**Age Group (Ref = 55+ Years)**								
	0					0.00	0.00	0.00	**0.00**
	1–4					0.87	0.53	1.41	0.56
	5–14					5.69	3.55	9.10	**0.00**
	15–34					3.87	2.32	4.89	**0.00**
	35–54					1.453	1.01	2.10	**0.05**
	**Model AIC**	**2114.75**				**161.96**			

Two models were fitted; in Model 1 analyzing age as a continuous variable and sex; in Model 2, both age and sex were analyzed as categorical variables. Model had better fit based on a lower AIC value than Model 1

Model 2 had a lower AIC therefore had better fit model, than Model 1. However, Model 1 does show statistically significant differences in the risk of dying from NCDs compared to communicable diseases for both males and females. Males had about 21% lower odds (than females) of dying from NCDs compared to dying from communicable diseases. Model 1 further shows that males (compared to females) have three times higher odds of dying from external causes than from communicable diseases. Model 1 further shows that with the increase in age, the risk of dying from NCDs or external causes (compared to dying from a communicable disease) also increases – in this case by about 2 % odds.

Model 2 results show that the odds of dying from an NCD (compared to a communicable disease) were higher for those aged 55 years and older compared with those aged less than 55 years. Although data in Table 4 shows that males had lower odds of dying from an NCD than a communicable disease when compared to the females, (as seen in Model 1) these results were found not to be statistically significant at 95% confidence level.

For external causes, Model 2 results show that males (compared to females) had higher odds (over twice as much) of dying from an external cause than from a communicable disease. Infants had almost zero risk (99.9% lower odds) of dying from an external cause than from a communicable disease when compared with the reference group. However, those aged 5–14 years had over five and half times higher odds of dying from an external cause than from a communicable disease. Similarly, those aged 15–34 years were more likely to die from external causes than from communicable diseases, while those aged 34–54 years were about 1.5 times likely to die from external causes compared with the reference group.

## Discussion

The civil registration system in Zambia, just as it is in many developing countries, is not fully functional and cause of death data from this system, are of limited public health utility. This largely arises from lack of medical death certification, especially for deaths that occur outside of hospitals. The reliance on lay reporting of the cause of death by relatives of the deceased and “authenticated” with the police reporting system, has in many times than not led to the production of unreliable and unusable cause of death data that has generally made it almost “impossible” to understand the disease burden the country is facing.

The utilization of VA in routine civil registration system can help provide a more reliable source for ascertaining the cause of death information for non-facility deaths [8, 28]. The VA implementation in Lusaka, based on the BID system, demonstrates that such a system can provide robust information to produce a more reliable mortality profile, with characteristics and patterns, for non-facility deaths. Combined with the cause of death information from health facility deaths, this system can produce a complete mortality profile for the city of Lusaka with a potential to produce more if escalated to the whole country. This however, is contrary to what [29] found in the application of VA to determine cause of death in rural Vietnam where the use of VA gave more precise cause of death than those reported at death registration.

VA was applied in routine civil registration in an urban district of Zambia. Results of this study demonstrated a high prevalence of external causes of deaths arising from road traffic accidents, homicides, poisoning and suicides, affecting mostly males in the ages 20–34 years. In the same way, most of the infectious diseases stemmed from HIV/AIDS-related OIs of pneumonia and tuberculosis, and from malaria and diarrhea diseases; half of them attributable to HIV/AIDS and related causes. These results further showed a high proportion of NCDs ranging from stroke to various kinds of cancers, such as prostate, leukemia/lymphomas, breast cancer and cervical cancer burdening mostly the population of Lusaka aged 35 years and older. The ratio of deaths from NCDs to that of deaths from communicable diseases is indicative of the country’s or an areas’ epidemiological transition position [30]. As observed in this study, the ratio of 0.77 (1 to 1.3), shows a higher burden of infectious diseases such as HIV/AIDS related and tuberculosis, but also with a large enough representation of NCDs, demonstrating clearly of the high burden of NCDs in Lusaka for non-facility deaths. Though our study was able to bring out the possible COD from external, infectious and NCDs, research conducted elsewhere revealed that VA tended to overdiagonse important causes such as diabetes, liver cancer, and tuberculosis, while undercounting deaths from HIV/AIDS, liver disease, essential renal and digestive system disorders [31].

Regression analysis suggest that the burden of the three broad disease groups differed by age and sex. External causes were highly prevalent among males between ages 20 and 34 years, while NCDs were more prevalent among those aged 55 years and older; with no observed sex differences. Despite communicable diseases burdening all ages and both sexes, HIV/AIDS and tuberculosis burdens were highest among those aged 20–54 years, while pneumonia was highest among infants and adults aged 55 years and older. Diarrhea and dysentery on the other hand mostly affected children under the age of 5 years. Results further showed that, compared to dying from a communicable disease, the risk of dying from an external cause increased by age and was highest between ages 5 and 34 years. While the risk of dying from an NCD also increased by age, compared to dying from a communicable disease, the risk of dying from an NCD was lower even in older ages (55+ years); implying that the risk of dying from a disease such as pneumonia outweighed the risk of dying from cancer in older ages.

The VA also captured a significant proportion of neonatal deaths, which are not so easy to detect in regular civil registration system since neonatal deaths are usually under-reported in less developed countries like Zambia were its considered culturally sensitive, especially outside health facilities. Though this is in agreement with what [32] found in their study on ascertaining causes of neonatal deaths using VA, they recommended that, with respect to neonatal deaths, additional methods to improve VA should be explored such as incorporating the use of a social autopsy to address issues of care seeking.

In the same way, the VA also recorded stillbirth, identified when the reported deceased neonate did not show any signs of life after birth.

These VA findings show important evidence on the broader applicability and utility of the VA method for improving cause-of-death information for non-facility deaths. This finding is in agreement with what [33] were they found that if VA can help close critical information gaps that exists in COD information and may guide policy development, implementation, evaluation and investment in routine information systems as well as data capturing. Nonetheless, caution must be taken since their application may have limitations to take into account. One important limitation of this application lies in the underlying uncertainty of the VA method arising from information bias in responses to the actual VA interview. This potential bias may arise if the respondent had less information about the deceased, especially if that particular respondent was not the primary care giver of the deceased. Results in this paper show that undetermined COD cases were lower for neonatal and child deaths than adult deaths. This could be a result of care givers providing scanty information or indeed not willing to provide all details regarding signs and symptoms for the deceased neonate or indeed child. It could also mean that relatives who brought the deceased neonate or child the mortuary were not primary care givers. In addition, a number of BIDs (mostly adults) are brought to mortuaries by the police (after being found dead) and therefore unaccompanied by any relative; such posses challenges to fully apply the VA to provide additional information for that particular death.

While VA is used globally by networks such as GBD and DHS [19, 20], the SmartVA algorithm does pose challenges as well. Although the algorithm was able to identify most individual causes of death, it was not able to identify all causes, owing to the aggregation of CODs into broader causes such as “other non-communicable diseases” or “other Injuries”. Such aggregations are not very informative especially if they are in the top-ten leading causes of deaths. Specificity if such condition is required, especially if the information provided is to be used for the civil registration system and/or for public policy. The methodological challenges non-specific underlying causes of death is a well know problem and continues to be a problem for population health scientist [21].

Finally, it should be noted that results of this demonstration are not to be generalized to represent the mortality profile for Lusaka; they only represent the mortality pattern of non-facility deaths for the reference period under this study.

## Conclusion

The application of the VA to the civil registration system specific to Zambian is an important step towards the availability and improvement of quality cause of death data for public health policy and decision-making. Demonstrations, such as this particular one supported by the Bloomberg Data for Health Initiative in Lusaka can produce more reliable cause-specific mortality fractions that the country needs to know its disease burden, especially for deaths that occur in communities and not attended to by health personnel. Our study has added to the body of knowledge about the application of verbal autopsy in a routine civil registration system in a country with a very poor civil registration system. More importantly, this study has presented leading causes of non-facility deaths in Lusaka and has assessed the age-and-sex and associated characteristics, which are key to understanding the general mortality profile.

## Data Availability

The datasets generated and/or analysed during the current study are not publicly available due to the sensitiveness of the data, however, may be made available from the corresponding author on reasonable request.

## Abbreviations

AIDS: Acquired Immunodeficiency Syndrome
AIC: Akaike Information Criterion
BID: Brought in Dead
CDs: Communicable Diseases
COD: Cause of Death
CRVS: Civil Registration and Vital Statistics
CSMF: Cause Specific Mortality Fractions
D4H: Data for Health
DRPC: Department of National Registration Passport and Citizenship
GBD: Global Burden of Disease
HIV: Human Immunodeficiency Virus
ICD: International Classification of Diseases
IHME: Institute for Health Metrics and Evaluation
NCD: Non-Communicable Disease
NCDs: Non-Communicable Diseases
ODK: Open Data Kit
PHMRC: Population Health Metrix Research Consortium
SPSS: Statistical Package for Social Scientist
UTH: University Teaching Hospital
VA: Verbal Autopsy
WHO: World Health Organisation
